# Oxysterol binding protein (OSBP) contributes to hepatitis E virus replication

**DOI:** 10.1186/s12985-024-02438-3

**Published:** 2024-07-22

**Authors:** Shaoli Lin, Peixi Chang, Shane Tsao, Abigail Aderinwale, Bhargava Teja Sallapalli, Jia He, Yanjin Zhang

**Affiliations:** 1 Molecular Virology Laboratory, VA-MD College of Veterinary Medicine, University of Maryland, College Park, MD USA; 2 National Cancer Institute, National Institute of Health, Bethesda, MD USA

**Keywords:** Hepatitis E virus (HEV), Oxysterol binding protein (OSBP), Helicase, Viral replication

## Abstract

Hepatitis E virus (HEV) is a positive-sense, single-stranded RNA virus and causes primarily acute self-limiting infections. The ORF1 of the HEV genome encodes a polyprotein around 190 kDa, which contains several putative domains, including helicase and RNA-dependent RNA polymerase. The HEV-encoded helicase is a member of the superfamily 1 helicase family and possesses multiple enzymatic functions, such as RNA 5′-triphosphatase, RNA unwinding, and NTPase, which are thought to contribute to viral RNA synthesis. However, the helicase interaction with cellular proteins remains less known. Oxysterol binding protein (OSBP) is a lipid regulator that shuffles between the Golgi apparatus and the endoplasmic reticulum for cholesterol and phosphatidylinositol-4-phosphate exchange and controls the efflux of cholesterol from cells. In this study, the RNAi-mediated silencing of OSBP significantly reduced HEV replication. Further studies indicate that the HEV helicase interacted with OSBP, shown by co-immunoprecipitation and co-localization in co-transfected cells. The presence of helicase blocked OSBP preferential translocation to the Golgi apparatus. These results demonstrate that OSBP contributes to HEV replication and enrich our understanding of the HEV-cell interactions.

**Supplementary Information:**

The online version contains supplementary material available at 10.1186/s12985-024-02438-3.

## Introduction

Hepatitis E virus (HEV) is a positive-sense, single-stranded RNA virus and causes primarily acute self-limiting infections. HEV infection can cause fulminant hepatic failure in infected pregnant women in South Asian countries, leading to a case-fatality rate of up to 30% [1–3]. HEV infection also causes substantial morbidity in pregnant women in high-income countries [4]. The infection can become chronic in immunocompromised individuals, including organ transplant recipients, leukemia, lymphoma, and HIV-infected patients [5, 6], and in patients beyond transplantation or HIV infection in high-income countries [7, 8]. HEV is a member of the genus *Paslahepevirus,* the family *Hepeviridae* [8–10]. There are eight genotypes in the species *Paslahepevirus balayani*. Genotypes 1 and 2 HEV strains are found only in humans; however, genotypes 3 and 4 are zoonotic with an expanded host range [8, 11, 12]. Genotype 1 and 2 HEV strains appear more virulent than genotypes 3 and 4 [10]. Genotypes 5 and 6 are found in wild boars, while genotypes 7 and 8 are found in camels [13]. A genotype 7 HEV strain was found in a patient with a chronically infected liver transplant who regularly consumed camel meat [14]. The HEV RNA genome contains three open reading frames (ORF), among which ORF1 encodes the non-structural polyprotein for viral RNA replication, ORF2 for the capsid protein, and ORF3 for a multifunctional protein [15, 16]. HEV is understudied, and its mechanistic insights into HEV-cell interactions remain largely unknown.

The ORF1 encodes a polyprotein (pORF1) around 190 kDa, which contains several putative domains: methyltransferase (Met), Y, papain-like cysteine protease (PCP), X (macro), helicase (Hel), and RNA-dependent RNA polymerase (RdRp) [6]. The Y domain is considered as an extension of Met and termed as an “Iceberg” region [17]. Helicases are grouped into six superfamilies on the basis of sequence homology in conserved motifs [18]. HEV helicase is a member of the superfamily 1 (SF1) helicases with all seven signature motifs (I, Ia, II, III, IV, V, and VI). It possesses multiple enzymatic functions, such as RNA 5′-triphosphatase activity involved in the cap formation, RNA unwinding, and NTPase activities, indicating that the protein may participate in viral RNA synthesis [19, 20].

Liver cells are essential for the regulation of cholesterol levels in the body [21, 22]. The liver synthesizes cholesterol and triglycerides (TG) for export to other tissues. It also eliminates cholesterol from the body by converting it into bile salts. In this process, various lipoproteins are involved in transporting cholesterol, among which low-density lipoprotein (LDL) and very-low-density lipoprotein (VLDL) mediate the cholesterol transportation from hepatocytes to the other cells [22]. The high-density lipoprotein (HDL) is responsible for the return of cholesterol to the hepatocytes [22, 23]. The entry of HEV into Huh-7 cells requires the presence of cholesterol [24]. During acute hepatitis E virus infection, serum TG and LDL are significantly higher than in healthy controls, while cholesterol and HDL are lower [25]. HEV infection of A549 cells leads to lower cholesterol [26]. Raising intracellular cholesterol via LDL or 25-hydroxycholesterol interferes with virus release. In chronically infected patients, TG, total cholesterol, and LDL levels are significantly lower after HEV infection, while there was no change in HDL level [26]. These data suggest that HEV infection-associated liver injury can interfere with lipid homeostasis.

During the dynamic lipid metabolism, the members of the OSBP (oxysterol binding protein)-related proteins (ORPs) family shuffle among the intracellular membranous web, transporting sterols between the cellular membrane and intracellular compartments. The family of ORPs consists of 12 members in mammals: OSBP, ORP1L, -2, -3, -4, -5, -6, -7, -8, -9, -10, and -11, all of which contain a core OSBP-related domain (ORD), and many also contain pleckstrin homology (PH) domains, transmembrane regions, ER-targeting FFAT (two phenylalanines in an acidic tract) motifs [27]. OSBP possesses a carboxy-terminal ligand-binding domain that binds a variety of lipids, such as phosphatidylinositol-4-phosphate (PI4P), cholesterol, and oxysterols [28]. OSBP functions to transport cholesterol from the ER to the Golgi apparatus and relocates PI4P from the Golgi to the ER [28, 29]. OSBP is present widely in membranes derived from ER vesicles, such as trans-Golgi, late endosomal, and mitochondrial membranes [30–32]. OSBP controls cholesterol/PI4P exchange at the ER-Golgi membrane contact sites. The PH domain of OSBP is responsible for the binding of the Golgi membrane as well as PI4P [33], while the FFAT motif binds to the vesicle-associated membrane protein-associated protein A (VAP-A) on the ER. The ORD binds both oxysterol and cholesterol [34]. The membrane tethering of OSBP is a dynamic balance process. Most OSBP exists in the cytoplasm in a soluble form in normal cells, with its PH domain masked by the C-terminal oxysterol binding domain. When exposed to a cholesterol-depleted environment or loaded with oxysterol, OSBP is triggered to translocate to Golgi [35].

The objective of this study was to determine the role of OSBP in HEV infection and elucidate the mechanism of the OSBP contribution. This paper presents data on the contribution of OSBP to HEV replication and the association of HEV helicase and OSBP. These results reveal the proviral role of OSBP in HEV infection and provide insights into the virus-cell interactions.

## Materials and methods

### Cells and viruses

Huh7.5.1 [36], HEK293T (ATCC® CRL-3216™), GP2-293 (Takara Bio USA, Inc., San Hose, CA), and HeLa (ATCC® CCL-2™) cell lines were maintained in the Dulbecco’s modified Eagle’s medium (DMEM) supplemented with 10% fetal bovine serum (FBS), 1 mM sodium pyruvate, 100 U/ml penicillin and 100 μg/ml streptomycin. The p6/Luc, an HEV replicon of the Kernow strain containing an insert encoding Gaussia luciferase reporter replacing the 5′ portion of ORF2, was used for in vitro transcription to generate HEV RNA for transfection [37]. The luciferase with a signal peptide is secreted out of the cells after synthesis.

### Plasmids

The helicase (Hel) domain (amino acid (aa) residues 1032-1276 in the pORF1 polyprotein) and methyltransferase (Met) domain (aa1-240 in the pORF1 polyprotein) from HEV Kernow-C1 strain (GenBank Accession Number: JQ679013) and helicase deletion mutants, D1 and D2, were cloned to the pCAGEN vector with an HA tag at the N terminus, as previously described [38, 39]. OSBP ORF clone (NM_002556) was purchased from OriGene Technologies (Rockville, MD) and was amplified by PCR for subcloning to the pCAGEN vector with an HA tag or FLAG tag at the N-terminus and pCDNA3-tdTomato vector, as described previously [38]. EYFP-GalT [40] (a gift from Jennifer Lippincott-Schwartz, Addgene Plasmid #11936) is used as a Golgi marker. The oligos for shRNA against OSBP (Table 1) were cloned to the pSIREN-RetroQ-ZsGreen vector as instructed (Takara). Primers used in this study are listed in Table 1. All plasmids constructed in-house were subjected to verification by DNA sequencing. The plasmid YFP-vp13 was described previously [41], and pVSV-G [42] was obtained from Addgene (a gift from Akitsu Hotta, Addgene plasmid # 138479).

**Table 1:** Primers used in this study

Primer^a^	Sequence (5′ to 3′)^b^	Target
KHel-F1	C*GAATTC*GGCTGCACTATCAGTCCTG	Helicase
KHelR2	G*CTCGAG*TTAGAAAAAATTATTGACAATCAC	Helicase
Helicase D1-R1	A*CTCGAG*TTACGCACCAGGGTTAGCGGCCT	Helicase D1
Helicase D2-F1	A*GAATTC*TTCGCGGCTTTCACACCTC	Helicase D2
OSBP-F1	T*CGTCTC*GAATTCGCGGCGACGGAGCTGAGAG	OSBP
OSBP-R1	C*CGTCTC*CTCGAGTCAGAAAATGTCCGGGCATGAGCT	OSBP
KMet-F1	C*GAATTC*ATGGAGGCCCACCAGTTC	Met
KMet-R2	G*CTCGAG*TTAGATCCACGCACGAAGTATG	Met
shOSBP-F1	*GATCC*GCATCTGAAAGCTAGTTCAGAATTCAAGAGATTCTGAACTAGCTTTCAGATGTTTTTT*G*	OSBP
shOSBP-R1	*AATTC*AAAAAACATCTGAAAGCTAGTTCAGAATCTCTTGAATTCTGAACTAGCTTTCAGATGC*G*	OSBP

^a^F: forward primer, R: reverse primer. All primers of helicase are based on the sequence of HEV Kernow-C1 P6 (GenBank accession# JQ679013)

^b^The italicized alphabets indicate restriction enzyme cleavage sites for cloning

### Transfection

Cells were transfected with plasmid DNA using jetOPTIMUS® DNA transfection Reagent (Polyplus transfection, New York, NY) according to the manufacturer’s instructions.

### Immunofluorescence assay (IFA)

IFA was carried out as previously reported [43] with primary antibodies against the HA tag (ABclonal, Woburn, MA) and the FLAG tag (Sigma-Aldrich, St. Louis, MO) and fluorescein-conjugated secondary antibodies: goat anti-rabbit IgG(H&L) Dylight 488, goat anti-mouse IgG(H&L) Dylight 549, and goat anti-mouse IgG(H&L) Dylight 649 (Rockland Immunologicals, Inc., Limerick, PA). SlowFade Gold antifade reagent containing DAPI (4=,6=-diamidino-2phenylindole) (Thermo Fisher Scientific, Waltham, MA) was used for coverslip mounting, followed by imaging with a Zeiss LSM800 confocal microscope. For quantifying the degree of co-localization of two fluorophores, Zeiss Zen imaging software was used to obtain the Pearson’s correlation coefficient (PCC): a value of 1 indicates perfect correlation, 0 for no correlation, and -1 for an ideal anti-correlation.

### OSBP silencing

GP2-293 cells were co-transfected with pSIREN-RetroQ-ZsGreen-shOSBP and pVSV-G plasmids at a ratio of 1:1. Forty-eight hours later, the culture supernatant was harvested and subjected to centrifugation at 10,000×*g* for 2 min to eliminate the cell debris. The cleared supernatant was filtered by 0.45 µm filter unit and added to Huh7.5.1 cells, followed by incubation for 3 days. Then, the cells were harvested for immunoblotting.

### Computational analysis of protein interaction

The interaction interface between HEV helicase and OSBP was modeled with AlphaFold 3 Webserver [44]. Full-length helicase (aa1-245) and OSBP (aa1-807) were uploaded for the modeling.

### Electroporation

The Huh7.5.1 cells in 6-well plates were detached with trypsin, rinsed with Opti-MEM twice, and resuspended in 200 μl Opti-MEM. HEV RNA was added to the tubes at 4 μg each and mixed gently. The mixture of cell suspension and RNA was then transferred to a 0.4-cm cuvette for pulsation in Gene Pulser X cell electroporation (Bio-Rad Laboratories, Inc., Hercules, CA) under the condition of 600 V, 1 pulse, and 0 intervals. The cells were then plated for culture.

### Luciferase assay

After the electroporation, samples of culture supernatant at 100 μl were taken from each well once every 24 h and frozen at − 80 °C. The same amount of fresh medium was then added to the culture for further incubation. Supernatant samples from the entire experiment were thawed together and assayed for luciferase activity using the Renilla luciferase assay system (Promega Corporation, Madison, WI) according to the manufacturer’s instructions. Briefly, supernatant samples were added to a 96-well black, flat-bottom microplate (Corning, Corning, NY), followed by the addition of the same amount of Renilla luciferase assay substrate and the detection of luminescence using a PerkinElmer 1420 Multilabel Counter. Three replicates were tested for each group. The luminescence was measured as relative luminescence units (RLUs) according to the manufacturer’s instructions.

### Co-immunoprecipitation (Co-IP)

Co-IP was conducted as described [45] with the antibodies against the HA tag (ABclonal) or the FLAG tag (Sigma-Aldrich). Protein A/G Magnetic Beads for IP (Bimake, Houston, TX) were used according to the manufacturer’s instructions. The Co-IP samples were subjected to Western blot analysis.

### Western blotting (WB)

Protein samples of cell lysate or Co-IP product were subjected to Western blot analysis as previously described [38]. The primary antibodies used in this study were rabbit anti-OSBP (Proteintech Group, Inc., Rosemont, IL), mouse anti-GAPDH (Santa Cruz Biotechnology, Inc., Dallas, TX), rabbit and mouse anti-HA tag (ABclonal), mouse anti-GFP (Biolegend, San Diego, CA), mouse anti-FLAG tag (Sigma-Aldrich), and mouse anti-β-tubulin (Sigma-Aldrich). The secondary antibodies used in WB were goat anti-rabbit IgG (H+L)-HRP conjugate and goat anti-mouse IgG (H+L)-HRP conjugate (Bio-Rad).

### Statistical analysis

Differences in indicators between treated samples, such as the Gaussia luciferase level between OSBP-depleted and control cells, were assessed by the Student *t-*test. A two-tailed *P* value of less than 0.05 was considered significant.

## Results

### Silencing of OSBP reduces HEV replication in Huh7.5.1 cells

**Fig. 1:**
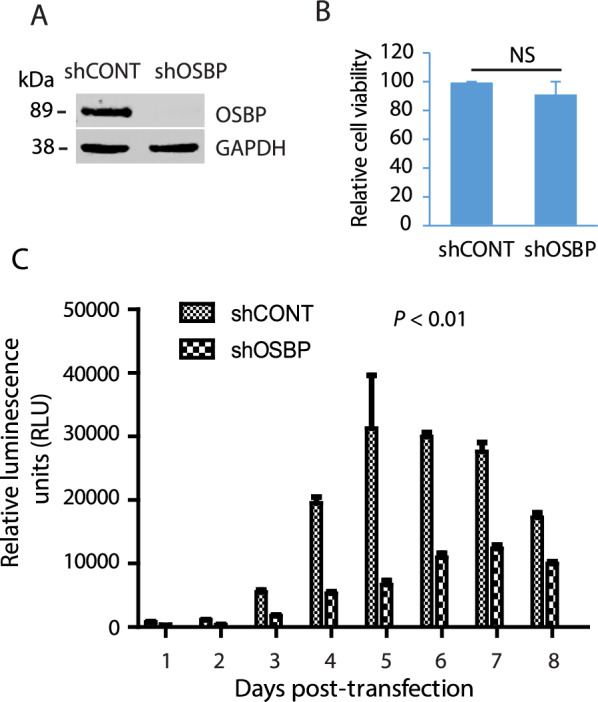
Silencing of OSBP impairs HEV replication in Huh7.5.1 cells. **A** Western blotting detection of OSBP in OSBP-depleted Huh7.5.1 cells. shCONT: control shRNA. shOSBP: shRNA against OSBP. The cells were transduced with retrovirus encoding shRNA and, three days later, harvested for WB. **B** Cell viability of Huh7.5.1 cells after OSBP silencing. The retrovirus-transduced cells were harvested for cell viability assay 3 days later. NS: no significant difference. **C** OSBP silencing leads to lower HEV replication. RNA of HEV P6/luc replicon was transfected into Huh7.5.1 cells with OSBP depletion. Culture supernatant was collected daily for luciferase detection for 8 consecutive days. Error bars represent the standard errors of three replicates of a representative experiment of three repeated experiments. The statistical difference (*P* < 0.01) was obtained from the analysis of group data between shCONT and shOSBP in all time points

To determine the role of OSBP in HEV replication, we conducted silencing of OSBP in Huh7.5.1 cells using a shRNA-based approach. The retrovirus encoding shRNA against OSBP (shOSBP) was transduced into Huh7.5.1 cells. A retrovirus encoding an irrelevant shRNA (shCONT) was included as a control in the transduction. The Western blotting (WB) results showed that shOSBP treatment led to a significant reduction of the OSBP protein level compared to shCONT (Fig. 1A). A cell viability assay was done 3 days after the transduction to determine whether the silencing of OSBP affects cell growth. The result showed that the silencing of OSBP had minimal effect on the cell growth of Huh7.5.1 cells (Fig. 1B).

The OSBP-silenced Huh7.5.1 cells were transfected with RNA from HEV p6/luc replicon. The cells transduced with shCONT were included in the transfection as a control. The expression of the luciferase reporter indicates the HEV replication level. Cell culture supernatant samples were collected daily for 8 consecutive days for luciferase assay. The results showed the silencing of OSBP in Huh7.5.1 cells led to lower HEV replication from 2 to 8 days post-transfection than the control (Fig. 1C). These results demonstrate that OSBP contributes to HEV replication.

**Fig. 2:**
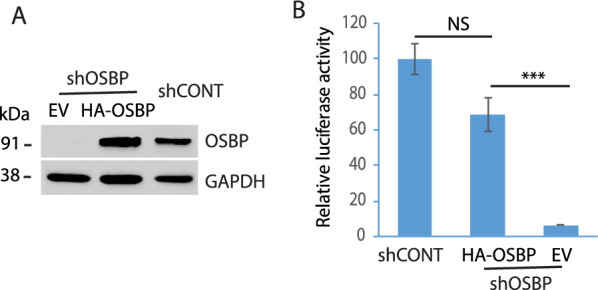
Ectopic expression of OSBP restores HEV replication in OSBP-depleted Huh7.5.1 cells. **A** WB of OSBP ectopic expression in Huh7.5.1 cells. The empty vector was included as a control. The Huh7.5.1 cells were transduced with retrovirus encoding shOSBP for 2 days and then transfected with HA-OSBP or empty vector (EV) for another 2 days. **B** OSBP trans-compensation restores HEV replication. The OSBP-depleted Huh7.5.1 cells with OSBP trans-compensation were electroporated with the RNA of the HEV replicon. The luciferase activity in the culture supernatant samples was tested 5 days later. ***: *P* < 0.001; NS: *P* = 0.074. Error bars represent the standard errors of three replicates of a representative experiment of three repeated experiments

To confirm the observation and exclude the possibility of off-target effect, we transfected the OSBP-silenced cells with OSBP plasmid. WB result showed the ectopic expression of OSBP in the OSBP-silenced cells (Fig. 2A). The cells with OSBP ectopic expression were transfected with HEV RNA. Luciferase assay result shows the restoration of HEV replication in the OSBP-silenced cells by the ectopic expression (Fig. 2B). The result demonstrates that the OSBP silencing was specific and substantiates the role of OSBP in HEV replication.

### HEV helicase interacts with OSBP and blocks its preferential translocation to the Golgi.

**Fig. 3:**
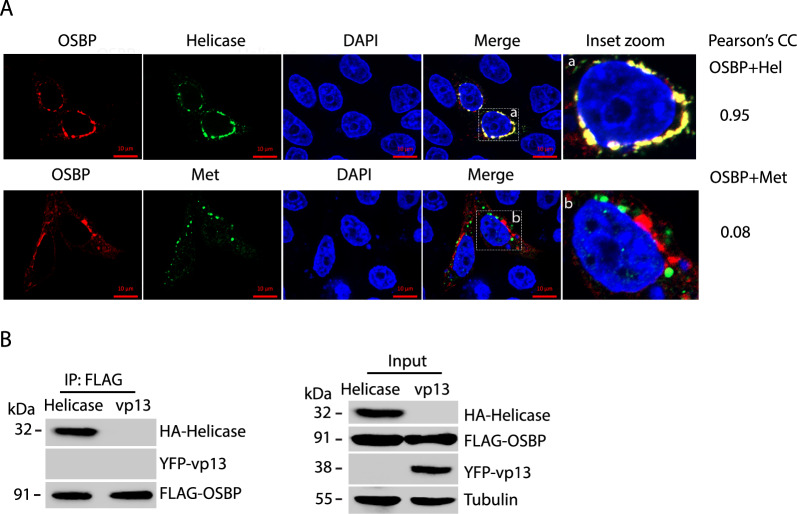
HEV helicase co-localizes and co-precipitates OSBP. **A** IFA shows the co-localization of helicase and OSBP. HeLa cells were co-transfected with plasmids of HA-helicase and FLAG-OSBP for 36 h. Co-transfection of OSBP and HA-Met plasmids was included as a control. The cells were fixed and stained with the rabbit antibodies against the HA tag and mouse antibodies against the FLAG tag. The bars in the lower right corner of the images denote 10 μm. Pearson’s CC (Pearson’s correlation coefficient) is shown on the right for the listed two proteins. **B** Co-IP of OSBP precipitates helicase. HEK293T cells were co-transfected with HA-helicase and FLAG-OSBP for 36 h. The cells were lyzed for Co-IP with FLAG antibody, followed by WB with HA tag antibody for the helicase. HEV ORF3 plasmid YFP-vp13 was included as a negative control

In order to identify the viral components potentially involved in the regulation of OSBP, HeLa cells were co-transfected with plasmids encoding OSBP and individual putative viral proteins, followed by immunofluorescence assay (IFA). Among the viral proteins tested, HEV helicase was found to co-localize with OSBP with Pearson’s correlation coefficient (PCC) of 0.95, while the PCC for Met and OSBP was 0.08 (Fig. 3A). Among the other HEV proteins, PCP and pORF3 were observed to partially co-localize with OBSP, and the rest had no noticeable co-localization (Supplemental Figure). To determine if HEV helicase interacts with OSBP, we transfected HEK293T cells with plasmids of FLAG-OSBP and HA-helicase. Co-Immunoprecipitation (Co-IP) of OSBP was conducted, followed by WB. The result showed that OSBP co-precipitated the helicase but not the YFP-vp13 control, which indicates that OSBP interacts with the HEV helicase (Fig. 3B). WB of cell lysate input was also done and the results showed the expression of these proteins.

**Fig. 4:**
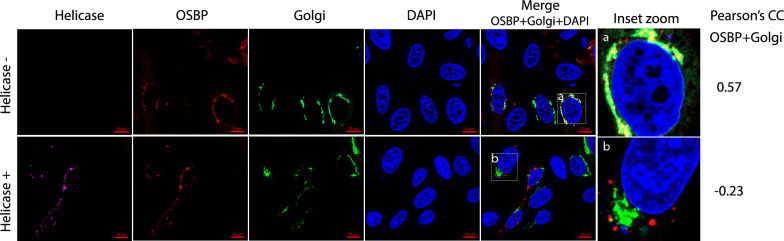
The HEV helicase blocks the preferential translocation of OSBP to the Golgi. OSBP is preferentially localized to the Golgi in HeLa cells (upper panel). The HEV helicase co-localizes with OSBP and blocks its preferential translocation to the Golgi (lower panel). The cells in the upper panel were transfected with Golgi marker EYFP-GalT and tdTomato-OSBP for 36 h. The cells in the lower panel were transfected with EYFP-GalT, tdTomato-OSBP, and HA-helicase for 36 h. The cells were probed with antibodies against the HA tag and anti-mouse IgG conjugated with Dylight A649. The bars in the lower right corner of the images denote 10 μm. PCC on the right indicates the co-localization of OSBP and Golgi marker in the presence and absence of HEV helicase

We speculated that helicase interaction with OSBP might interfere with its subcellular localization. To test this speculation, we transfected HeLa cells with plasmids of EYFP-GalT (Golgi marker), tdTomato-OSBP, and HA-helicase. IFA with antibody against the HA tag was conducted to determine the helicase expression. The results showed the preferential localization of OSBP to the Golgi (Fig. 4), which is consistent with the previous report [46]. However, the presence of the helicase blocks the preferential translocation of OSBP to the Golgi (Fig. 4). The change in OSBP localization suggests HEV may recruit OSBP via the helicase, which may exploit the OSBP functions during HEV infection.

### Helicase interacts with OSBP via their C-terminal domains

**Fig. 5:**
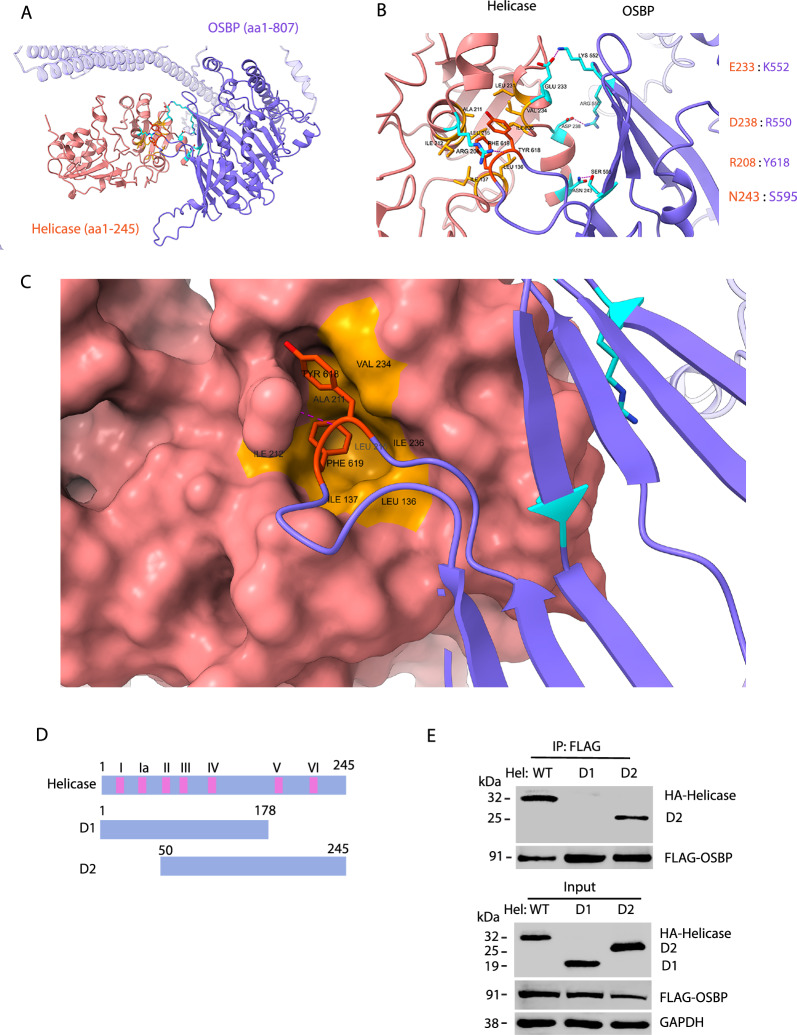
OSBP interacts with the C-terminal domain of the HEV helicase. **A** The interaction model between helicase and OSBP is predicted by Alphafold3. The C-terminal lobes of both proteins are involved in the interaction. **B** Zoom view of the interaction interface of helicase and OSBP. The residues for the four pairs of hydrogen bonds are labeled on the right. **C** At the predicted binding site, residues 618YF619 on OSBP are inserted into a hydrophobic pocket on the C-terminal lobe of HEV helicase. **D** Diagram of the HEV helicase truncations. Numbers above the bars indicate the helicase amino acid residues. The pink color bars indicate the seven helicase motifs. **E** OSBP co-precipitates helicase deletion construct D2. HEK293T cells were co-transfected with FLAG-OSBP and helicase truncations tagged with the HA. The cell lysates were harvested at 36 h post-transfection for Co-IP with FLAG antibody, followed by WB with HA tag antibody

To further analyze the interaction interface between HEV helicase and OSBP, we modeled the OSBP:Helicase complex with AlphaFold 3 Webserver [44]. The predicted complex structure indicates the C-terminal lobes from both OSBP and HEV helicase contribute to the interaction (Fig. 5A, B). At the predicted binding site, residues 618YF619 on OSBP are inserted into a hydrophobic pocket on the C-terminal lobe of HEV helicase, with nearby residues forming hydrogen bonds and salt bridges as labeled (Fig. 5B, C). In order to assess the interaction experimentally, we constructed two deletion constructs of helicase: D1 and D2 (Fig. 5D). The helicase-D1 includes the first five conserved motifs, and helicase-D2 contains all but the first two motifs. HEK293T cells were transfected with plasmids of FLAG-OSBP and HA-helicase. Co-IP with FLAG antibody was conducted. The result showed OSBP co-precipitated the helicase mutant D2 but not the D1 (Fig. 5E), suggesting OSBP might interact with the helicase’s C-terminal domain.

## Discussion

Our data demonstrate that OSBP contributes to HEV replication. OSBP silencing led to a significant reduction in the replication of an HEV replicon. Our results show that the HEV helicase is co-localized with OSBP, interacts with OSBP, and blocks its preferential translocation to the Golgi in co-transfected cells. Further analysis indicates the interaction between these two proteins occurs possibly via their C-terminal lobes. These results suggest that HEV may recruit OSBP to assist viral replication.

The OSBP silencing did not affect the cell viability much but significantly inhibited HEV replication. The possible reason for the minimal effect on cell viability is that the cells reaching confluency might not rely on OSBP for maintenance or other ORPs complement the OSBP loss under cultured conditions. However, the OSBP silencing reduces HEV replication. OSBP silencing possibly affects HEV RNA replication as the HEV p6/luc replicon was used, and the luciferase is expressed from the subgenomic RNA. The lower luciferase yield indicates a lower level of the subgenomic RNA that is produced by HEV RdRp. The proviral role of OSBP in HEV replication was further confirmed by the trans-complementation of OSBP in the cells with OSBP silencing. This ectopic expression of OSBP restored the HEV replication, which also excludes the possibility of an off-target effect of the shRNA silencing.

Among the HEV proteins, the helicase was discovered to co-localize with OSBP and interact with OSBP. There are multiple motifs on helicase, most of which (Ia, III, IV, V, and VI) possess nucleic acid or NTP binding activities [20]. The computational modeling of OSBP interaction with helicase predicted that the C-terminal lobes of both helicase and OSBP interact. The co-IP result indicates that OSBP interacts with the C-terminal region of the helicase.

To further examine how the virus regulates the OSBP activity, we did transient expression of OSBP and the HEV helicase in HeLa cells and found that the helicase blocked the preferential translocation of OSBP to the Golgi, suggesting that HEV may recruit OSBP via the helicase. Host lipid homeostasis is often vital for virus infection, and many lipid-associated proteins are required for virus replication [47, 48]. For instance, apolipoprotein B, which is associated with the maturation and secretion of LDL, plays an essential role in HCV particle exocytosis [49]. Upon Coxsackievirus B3 (CVB3) infection, the viral 3A protein localizes to secretory organelle membranes. It recruits GBF1/Arf1, thereby enhancing the accumulation of PI4KIIIB, which further catalyzes the production of uncoated PI4P-enriched structures adjacent to ER exit sites [50].

During the infection by some viruses, OSBP is exploited to facilitate the trafficking of lipid components to promote viral replication. For example, OSBP is a downstream effector of phosphatidylinositol 4-kinase alpha (PI4KIIIα) for HCV replication, aiding in the cholesterol trafficking in the viral-induced membranous web [51, 52]. The depletion of OSBP decreases the replication of HCV. Interestingly, complete depletion of OSBP reduces both intracellular and extracellular HCV viral RNA. However, partial depletion of OSBP affects only the accumulation of extracellular HCV RNA, which suggests that OSBP should be involved in the assembly and release of infectious HCV virions [51]. In Aichi virus replication, OSBP is recruited to the Aichi virus replication complex, possibly for constructing the organelle membrane [53]. In other circumstances, the substrate of OSBP can combat virus infection. For instance, the existence of oxysterol can inhibit HBV replication in both DNA and protein levels in hepatocytes [54]. Our results suggest that HEV recruits OSBP to benefit the virus replication. Further research is needed to study the mechanism of the proviral role of OSBP in HEV replication.

HEV helicase possesses RNA 5′-triphosphatase, RNA unwinding, and NTPase activities, which are thought to be involved in viral RNA synthesis [19, 20]. In addition to these enzyme activities, the HEV helicase was found to interact with multiple cellular proteins identified via yeast two-hybrid analysis [55]. Some of these proteins are involved in metabolic and biological processes. Our data shows that the helicase interacts with OSBP and changes its preferential localization, which might affect cellular cholesterol homeostasis. A previous study indicates that HEV infection reduces cholesterol levels in A549 cells and that increasing cholesterol levels in cultured cells reduces HEV release [26]. HEV infection in patients reduces serum TG, total cholesterol, and LDL levels [26]. Therefore, the helicase interaction with OSBP and changing its preferential localization might contribute to the cholesterol modulation in HEV-infected cells and patients.

This study’s limitations are that a luciferase-based HEV replicon was used and that the OSBP interaction with helicase was not performed in hepatocytes. Future studies using full-length HEV RNA transfection or HEV infection are needed to confirm the proviral role of OSBP and its interaction with pORF1 or helicase since both ORF2 and ORF3 products are involved in HEV assembly and release. The full-length HEV replicons with HA or V5-tagged ORF1 in the HVR [56, 57] appear to fit studies to determine the OSBP and pORF1 interaction.

In conclusion, this study shows that OSBP contributes to HEV replication and that the HEV helicase interacts with OSBP and blocks OSBP preferential translocation to the Golgi apparatus. The results shed light on the HEV recruitment of the lipid regulator OSBP and improve our understanding of the virus-cell interactions.

### Supplementary Information

Supplementary Material 1.

Supplementary Material 2.

## Data Availability

All data generated or analyzed in this study have been provided within the article and supplementary information.
