# Supplementary material for: Oxysterol binding protein (OSBP) contributes to hepatitis E virus replication
Source: Virol J. 2024 Jul 22;21:161. doi: 10.1186/s12985-024-02438-3 (PMC11265327; doi:10.1186/s12985-024-02438-3)
Supplement: Supplementary file 1 — Supplementary Material 1. [file 12985_2024_2438_MOESM1_ESM.docx]

Figure legend for supplemental figure

IFA shows the HEV proteins and OSBP. HeLa cells were co-transfected with FLAG-OSBP and HA-tagged HEV proteins for 36 h. The cells were fixed and probed with a rabbit antibody against the HA tag and a mouse antibody against the FLAG tag. The bars in the lower right corner of the images denote 10 μm. The HA-tagged PCP domain (aa433-592 in the pORF1 polyprotein of Kenow-C1 strain (GenBank Accession Number: JQ679013) ), the Macro domain (aa857-1014 in the pORF1 polyprotein) and RdRp (aa1279-1765 in the pORF1 polyprotein) were used in the transfection.
