# Supplementary figures and images for: Oxysterol binding protein (OSBP) contributes to hepatitis E virus replication
Source: Virol J. 2024 Jul 22;21:161. doi: 10.1186/s12985-024-02438-3 (PMC11265327; doi:10.1186/s12985-024-02438-3)

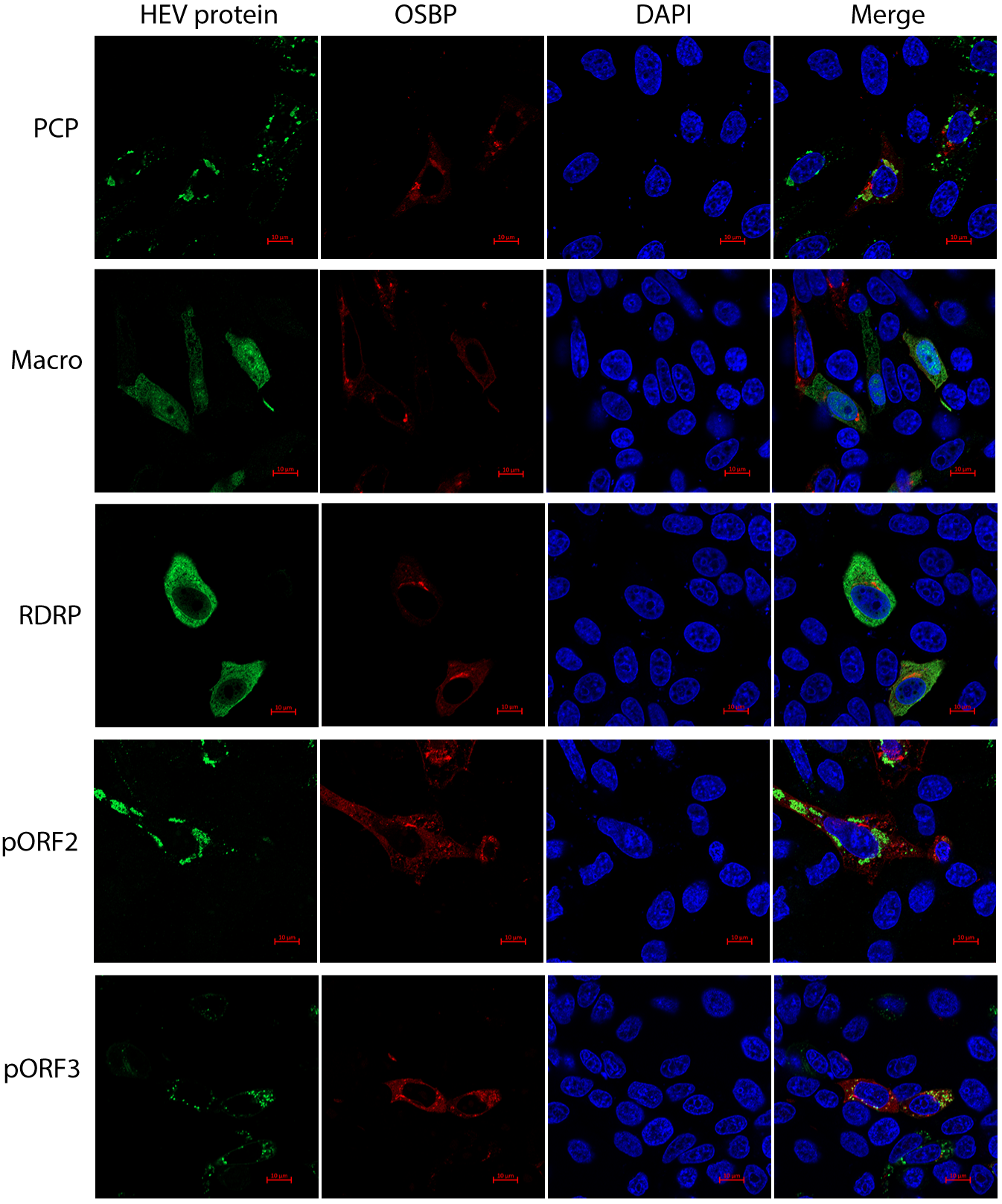

Supplement: Supplementary file 2 — Supplementary Material 2. [file 12985_2024_2438_MOESM2_ESM.tif]
